# Distinct molecular profile of the chick organizer as a stem zone during axial elongation

**DOI:** 10.1098/rsob.240139

**Published:** 2024-07-03

**Authors:** Timothy R. Wood, Iwo Kucinski, Octavian Voiculescu

**Affiliations:** ^1^ Department of Physiology, Development and Neuroscience, University of Cambridge, Cambridge CB2 3DY, UK.

**Keywords:** axial elongation, stem zone, organizer, chick

## Abstract

The vertebrate organizer plays a crucial role in building the main (antero-posterior) axis of the embryo: it neuralizes the surrounding ectoderm, and is the site of emigration for cells making axial and paraxial mesendoderm during elongation. The chick organizer becomes a stem zone at the onset of elongation; it stops recruiting cells from the neighbouring ectoderm and generates all its derivatives from the small number of resident cells it contains at the end of gastrulation stages. Nothing is known about the molecular identity of this stem zone. Here, we specifically labelled long-term resident cells of the organizer and compared their RNA-seq profile to that of the neighbouring cell populations. Screening by reverse transcription-polymerase chain reaction and *in situ* hybridization identified four genes (*WIF1*, *PTGDS*, *ThPO* and *UCKL1*) that are upregulated only in the organizer region when it becomes a stem zone and remain expressed there during axial elongation. In experiments specifically labelling the resident cells of the mature organizer, we show that only these cells express these genes. These findings molecularly define the organizer as a stem zone and offer a key to understanding how this zone is set up, the molecular control of its cells’ behaviour and the evolution of axial growth zones.

## 1. Introduction

The anterior–posterior axis of all vertebrate embryos is formed by a combination of two mechanisms: direct specification of anterior structures and sequential addition of material generated by posterior growth zones. Both processes involve a small region of the gastrulation site, the organizer. In amniotes, gastrulation takes place along the primitive streak (PS), with the organizer (node) at its tip. In the chick embryo, it has been shown that the organizer (Hensen’s node) acts in distinct ways during gastrulation and elongation stages.

**Figure 1.**
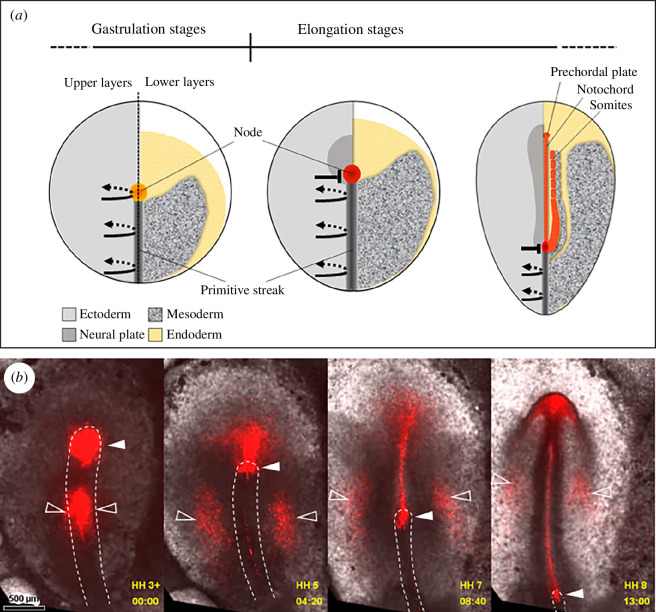
The chick organizer acts as a stem zone, generating axial and paraxial mesoderm during elongation. (*a*) The diagram summarizes the existing fate maps. Embryos at successive stages are shown in a split view, with the upper layer on the left side and the lower layers on the right side. Recruitment of epiblast cells to the primitive streak (black curved arrows) ceases at the level of the organizer at the initiation of elongation stages (black block arrow). (*b*) Embryo with the organizer and mid-primitive streak fluorescently labelled (red, open arrowheads), showing that the organizer retains resident cells that generate axial and paraxial structures (solid arrowheads), while all cells in the rest of the primitive streak emigrate away from it. Frames from electronic supplementary material, video S1.

During gastrulation, the organizer (like the rest of the PS) constantly changes its cellular composition. The epithelial cells at the PS undergo epithelial-to-mesenchymal transition (EMT) and ingress into lower layers; the constriction of their apical surfaces causes a pull on the cells in the neighbouring epiblast into the PS, which are in turn induced to undergo EMT [1,2]. At gastrulation stages, the organizer mainly produces gut endoderm [3,4]. A sharp transition marks the end of gastrulation and the beginning of axis elongation (figure 1; electronic supplementary material, video S1): the organizer stops recruiting cells [5] as it starts laying down its derivatives in head-to-tail sequence (head mesoderm, notochord and somitic mesoderm) [6]. Also, at the beginning of axial elongation, the ectoderm close to the organizer is stably neuralized. The neural territory anterior to the organizer is specified as the brain, while the regions on the sides of the organizer become growth zones that sequentially generate the caudal hindbrain and the spinal cord [7,8].

Remarkably, the organizer generates all its derivatives from the few cells it contains at the initiation of axial elongation. Single-cell labelling experiments have shown the organizer contains long-term residents capable of self-renewal, which also generate descendants contributing to head mesendoderm, and axial and paraxial mesoderm [9–11]. Here, we refer to the long-term resident cells in the anterior PS that generate descendants populating the axial and paraxial mesendoderm as the mesodermal stem zone (MSZ).

The molecular mechanisms setting up the organizer at gastrulation are well understood and conserved across vertebrates [12,13]. In contrast, nothing is known about the molecular and cellular mechanisms controlling the maintenance and function of the organizer during axial elongation (MSZ). At the start of axial elongation, the organizer stops expressing genes associated with gastrulation stages and also loses its morphological definition, its edges becoming indistinguishable from the surrounding neural ectoderm. The MSZ later becomes incorporated into the tailbud.

We therefore set out to identify genes specifically associated with the MSZ axial elongation stages. To this end, we specifically labelled the MSZ and compared its transcriptome profile with that of adjoining regions. Next, we performed expression screenings by reverse transcription-polymerase chain reaction (RT-PCR) and *in situ* hybridization for transcripts of higher expression in the MSZ. This led to the identification of four genes (*WIF1*, *PTGDS*, *ThPO* and *UCKL1*) that are upregulated in the organizer at the start of elongation stages and remain expressed in the MSZ as the axis is laid down. Graft experiments show that the expression of these genes is specific to resident cells of the MSZ. Most remarkably, these genes code for molecules of unrelated classes that are not known to be co-expressed in any other tissue. This set of genes now offers an unambiguous definition of the descendants of the organizer as a stem zone and can be used to distinguish it from adjacent stem cell populations. We discuss their possible functional significance and their use in evo-devo analysis of stem populations fuelling axial elongation.

## 2. Results

### 2.1. A unique molecular profile of the mesodermal stem zone

**Figure 2.**
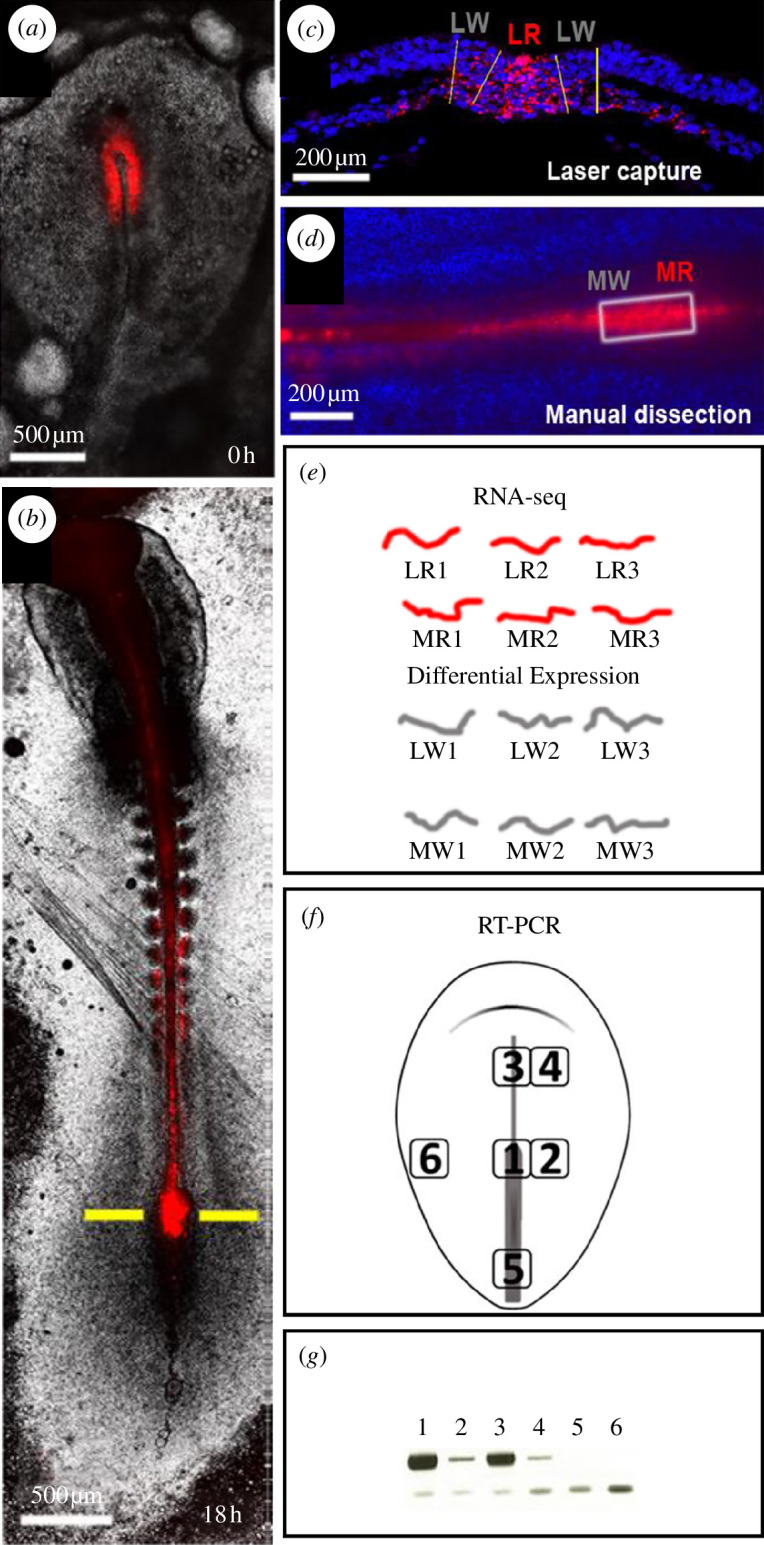
Method for the molecular profiling of organizer resident cells. (*a, b*) On fluorescent labelling (red) of the organizer at the end of gastrulation (*a*) allows the selective marking of its resident cells during elongation stages (*b*). (*c*) Thin section at the level of the anterior primitive streak (yellow line in (*b*)), showing the labelled and unlabelled collected by laser microdissection. (*d*) Region containing the anterior primitive streak and surrounding regions, used for the manual dissection of labelled and unlabelled cells. (*e*) Diagram of RNA-seq samples used for differential gene counts. (*f*) Embryonic regions excised used for the RT-PCR screening. (*g*) Exemplar of an RT-PCR (*ThPO*) showing localized expression in the anterior primitive streak and its derivatives. Lower bands are primers; upper bands are PCR products.

**Figure 3.**
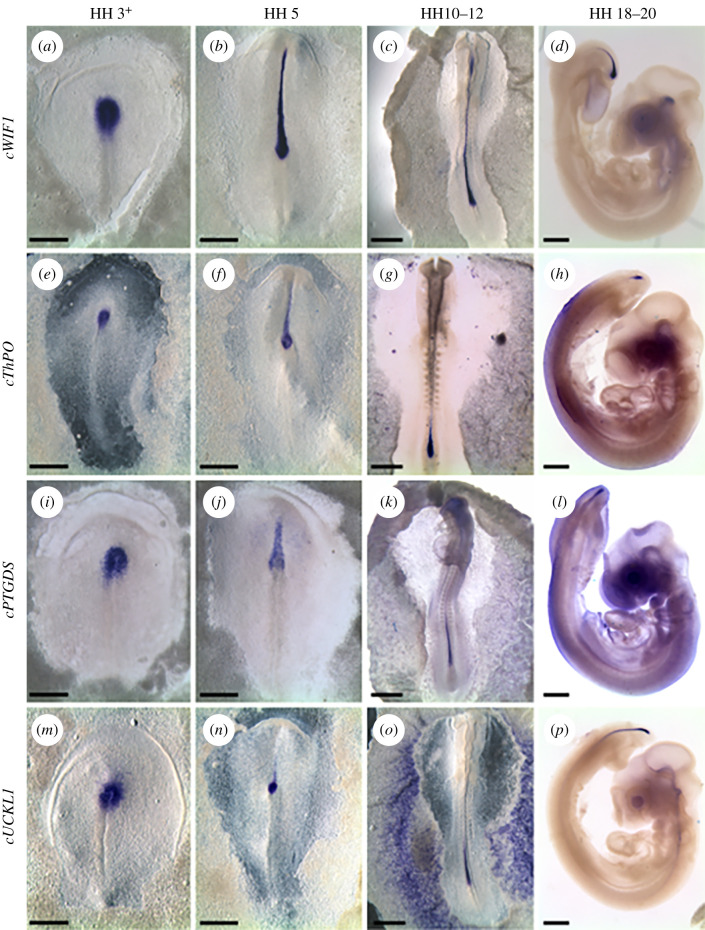
Four genes are expressed in the anterior primitive streak during the elongation stages. *In situ* hybridization for chick *Wif1*, *ThPO*, *PTGDS*, *UCKL1*. Scale bars, 500 µm (*a–c, e–g, i–k, m–o*), 1 mm (*d, h, l, p*).

We sought to identify genes specifically expressed by the organizer as it becomes a MSZ during elongation and distinguish it from the accompanying stem zones fuelling the extension of the neural plate. To this end, we devised a method of labelling resident organizer cells during axial elongation (figure 2*a,b*). We fluorescently labelled the organizer cells with a lipophilic dye (CM-DiI), just before it becomes a stem zone and the embryo starts elongating (fully extended PS, late stage HH 3^+^; see figure 2*a*). Labelled embryos were then allowed to develop for 16–24 h (figure 2*b*). In order to obtain transcription profiles of MSZ cells and select the transcripts whose expression does not change over time, cells were collected from embryos at a range of developmental stages, between 8 and 10 somites. Two separated cell populations were collected: (i) fluorescently labelled cells remaining in the anterior PS (‘R’ in figure 2*c,d*) and (ii) adjacent non-labelled cells in the neural plate or paraxial mesoderm (‘W’ in figure 2*c,d*). We used two methods to achieve this: laser capture on transverse sections through the node (figure 2*c*) and manual dissection of fluorescent and non-fluorescent cells from the region of the anterior streak (figure 2*d*). We found each technique presented complementary advantages and drawbacks (see §5). While manual dissection produces the best RNA quality (RIN = 10), it is relatively coarse. Laser capture offers more precise separation between populations, but the quality of the RNA is lower (RIN values around 7). For each method, three pairs of biological replicates were deep sequenced and compared (figure 2*e*; electronic supplementary material, table S1). We selected 93 transcripts that consistently showed higher gene counts in anterior PS resident cells than in neighbouring cells (electronic supplementary material, table S2). To examine their expression in the embryo, we first used RT-PCR on cDNA extracted from six different locations that included all embryonic layers (figure 2*f*): the anterior PS, the anterior midline containing some of the organizer derivatives, two neural domains (caudal and anterior), the caudal PS and the non-neural ectoderm. This initial screen indicated that 32 transcripts are present only in cDNA from the anterior region of the PS and the anterior midline (electronic supplementary material, table S2). These transcripts were further examined by *in situ* hybridization. Markers of MSZ cells should fulfil the following criteria: (i) not be expressed during gastrulation; (ii) be upregulated only in the anterior PS at the initiation of axial elongation; and (iii) retain their expression at this location throughout elongation. We found four genes that meet these criteria: *cWIF1*, *cThPO*, *cPTGDS* and *cUCKL1* (figure 3). The expression patterns of these genes in the anterior PS correlate with the location of the MSZ cells. However, at the onset of their expression (the end of gastrulation stages), the anterior PS contains several resident populations as well as transient cells, which occupy overlapping domains. At subsequent stages, the organizer also loses its morphological boundaries, and it is difficult to distinguish it from the neighbouring growth zones of the neural plate. We therefore set out to test more accurately how specific the expression patterns of the newly identified genes are for the long-term resident cells of the MSZ.

### 2.2. Resident node cells retain expression of mesodermal stem cells

**Figure 4.**
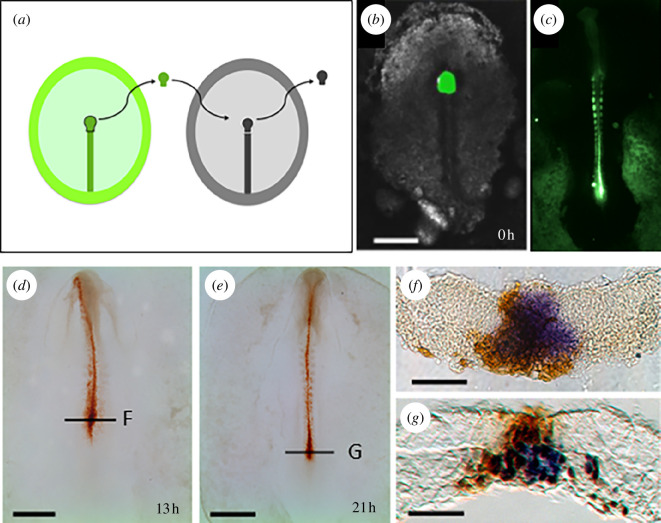
Specific expression of the identified genes in the organizer resident cells. (*a*) Homotopic and isochronic graft of a labelled anterior primitive streak to label all the resident cells in the organizer. (*b*) Example of grafted embryo, with fluorescently labelled node in green. (*c*) The operated embryo after 24 h of culture. (*d, e*) Examples of grafted embryos at different developmental stages, processed by immunohistochemistry; labelled cells are in brown. (*f, g*) Grafted embryos were processed for both *in situ* hybridization (*cWif1*, purple) and immunohistochemistry (brown). Thin sections at the levels are indicated in (*d, e*).

To test whether these genes are specific for the long-term resident cells of MSZ, all the cells in the anterior streak (both transient and long-term residents) need to be labelled at the initiation of elongation. Transient cells are then allowed to emigrate during subsequent stages, thus leaving the resident cells as the only labelled population. Marking all the cells of the organizer was achieved by homotopic and isochronic grafts of the anterior PS region, from fluorescently labelled embryos into unlabelled hosts, at the initiation of elongation. Embryos were then allowed to develop for 18–24 h to stages 5–15 somites (figure 4*a–c*). Immunohistochemistry (figure 4*d,e*) was employed to evidence all the cells bearing the fluorescent marker, and the expression pattern of the newly identified genes was assessed by *in situ* hybridization. In sections at the level of the anterior PS, we could identify only double-positive or double-negative cells (figure 4*f,g*; electronic supplementary material, video S2). These results show that the genes identified here are bona fide markers of the resident cell population constituting the MSZ.

## 3. Discussion

Single-cell lineage analyses [9,10] and retrotransplantations [11,14] previously showed the presence of a mesodermal stem cell population at the mature chick organizer during elongation; similar results were obtained in mice [6,15–20]. It is yet unknown how many actual mesodermal progenitors exist, how they cycle, how they are spatially organized and what constitutes their niche. In this study, we do not distinguish between these cell populations and report a molecular profile of cells residing in the descendants of the mature organizer, irrespective of their role. For this reason, we refer to the collection of these cells as a ‘zone’. These long-term residents PS remain cohesive and occupy the anterior part of the PS during retraction (accompanied by axis elongation). All cells in this zone appear to express the four genes identified here. It is however possible that some differences in the expression levels between cells are obscured in histological sections. Future investigations using more cellular resolution are needed, combined with lineage analyses, to examine whether specific subpopulations of cells express these genes differently.

On the lateral edges of this zone, very few marked cells seem not to express these genes (see figure 4*f,g*; electronic supplementary material, video S2). These are probably cells that emigrate out of the stem zone to populate the presomitic mesoderm. We have also observed in most embryos a few marked cells scattered in the PS just posterior to the MSZ (electronic supplementary material, video S2). At present, we do not know the significance of these cells, which may be brought out of the MSZ by the cellular rearrangements within the PS. These cells do not express the genes demarcating the MSZ.

The distinct molecular signature defining the chick organizer as a stem zone during elongation stages now opens up a number of avenues. This is a novel set of genes, with only *ThPO* previously reported as associated with the mature organizer, using a different and complementary approach[21].

## 4. Opening up

First, this set of genes can be used to rigorously distinguish between resident organizer cells (MSZ) and adjacent neighbouring stem populations (caudal neural plate and neuro-mesodermal bipotent progenitors).

Second, it is now possible to explore the molecular mechanisms regulating MSZ cell behaviours. The four genes identified here are specific markers of the MSZ (*WIF1*, *PTGDS*, *ThPO* and *UCLK1*) and code for proteins of diverse classes and functions. Their known functions immediately suggest several hypotheses. WIF1 is an inhibitor of both canonical and non-canonical Wnt pathways [22], which are involved in regulating multipotentiality in other stem cell populations. The prostaglandin synthase PTGDS [23] has been implicated in controlling EMT [24,25] in other cellular contexts. It has also been shown to trap and/or transport retinoids [26], independently of its catalytic function. This suggests that PTGDS may be involved in protecting the MSZ cells from the action of retinoids produced in more anterior regions. Thrombopoietin (ThPO) controls proliferation in haematopoietic niches [27,28], and UCKL1 in blast transformation [29,30]; thus, these genes may be involved in controlling the cell cycle progression of MSZ progenitors.

Third, the regulation of these genes offers a key to investigate how the mature organizer becomes a stem zone. To our knowledge, the four genes found here are not co-expressed in any other tissues, and it seems unlikely that any of them directly regulates the expression of the others. Finding the gene regulatory network of these genes will shed light on the molecular mechanisms responsible for the acquisition of stem cell properties by the organizer. Moreover, these markers can be used to understand what induces the stemness of the organizer at the end of gastrulation stages. Is this induced by signals emanating from neighbouring structures at the beginning of elongation (e.g. more posterior PS, neural plate), or does it result autonomously from the temporal progression of the organizer?

Finally, how evolutionarily conserved is this signature of the MSZ? In all vertebrate and most invertebrate embryos, the long axis is laid down through a combination of direct specification and posterior growth zones. It is still debated what the relative contributions of these mechanisms are and how evolutionarily conserved growth zones are. Examining the expression of these genes’ homologues in other vertebrates will help to identify the mesodermal stem cells, how these cells are arranged with respect to other progenitors and the relative contribution of growth zones to axial elongation across vertebrates.

## 5. Material and methods

Fertilized eggs of wild-type hens (Bovans Brown) were obtained from WinterEgg Farm (Thriplow, Herts, UK).

### 5.1. Embryo culture

Fresh, fertilized hen’s eggs were kept for up to one week at 15°C until use and incubated at 38°C for the embryo to reach the desired stages. Embryos were explanted and manipulated in Pannett-Compton saline and cultured using New’s technique [31], as modified and described previously [32].

### 5.2. Fluorescent labelling

Working dilutions of lipophilic CM-DiI (Invitrogen; cat. no. C7001) were prepared fresh by adding 1 µl of stock solutions (0.5% w/v in ethanol, kept at −20°C) to 9 µl of aqueous sucrose solution (6% w/v) pre-warmed to 37°C. Finely drawn capillaries, with a tip broken to a bore of about 1–2 µm, were used to deposit warm solution close to the epiblast of embryos kept in saline.

### 5.3. Microscopy

Time-lapse epifluorescence movies were acquired with a PlanApo N 2x/0.08 lens on an Olympus IX71 wide-field microscope, fitted with a fluorescence excitation source (CoolLed pE-2) and appropriate emission optics, motorized stage (Prior) and Hamamatsu C8484 camera controlled by HCImage software. The system was enclosed in a Perspex box, one wall of which is the top of a Marsh Automatic Incubator (Lyon, USA) to provide thermoregulation and air circulation.

### 5.4. Whole node transplantations

Donor embryos were fluorescently labelled by CMFDA (Invitrogen, cat. no. C2925) by bathing whole for 1 h at 38°C, in Pannett-Compton solution containing CMFDA diluted 1 : 250 from the stock solution (10 mM in DMSO, kept at −20°C). They were then transferred three times for 10 min each in fresh Pannett-Compton saline to remove excess dye before having their nodes excised. The nodes were excised from unlabelled receiving embryos, prepared in New culture; the labelled nodes were aspirated and transferred with the aid of a P2 pipette adjusted to 0.2 µl.

### 5.5. Microsurgery

Surgical manipulations were done using fine tungsten needles sharpened by electroelution [33]. For RNA-seq (figure 1*h*), the region containing the resident, fluorescently labelled cells in the mature organizer was first excised whole from the embryo, which was then subdivided into the brightly fluorescent regions of the anterior PS and the faintly or non-labelled regions. Operated embryos were kept at room temperature for 2–4 h, conditions under which embryos are in developmental diapause but healing can take place, and then placed in a humidified box at 38°C and allowed to grow.

### 5.6. Gene expression analyses

RT-PCR reactions (figure 2*f,g*) were done using Phusion High-Fidelity DNA Polymerase (ThermoFisher, cat. no. F553L) on cDNA generated from five nodes dissected at stages HH 4–7. The list of primers used is given in electronic supplementary material, table S2. The PCR fragments were gel-purified and sequenced to check that their sequence corresponded to the intended transcript, and DIG-RNA probes were synthesized using the SP6 promoter included in the reverse primers, as described in GEISHA. *In situ* hybridizations were performed using the methods described earlier [34].

### 5.7. RNA-seq

Total RNA was column-extracted from freshly dissected or laser-captured material using RNeasy Micro Kit (cat. no. 74004; Qiagen), with a yield of 1–8 ng per sample. Several fixation methods were compared, of which we found methacarn (methanol–chloroform–acetic acid, 6 : 3 : 1 volume proportions) to be most suitable in terms of morphology preservation and quality of extracted RNA. Embryos with the mature organizer fluorescently labelled were grown *in vitro* (see above). At the end of incubation, the glass ring with the vitelline membrane supporting the embryo was lifted from the culture dish. The membrane and embryo were quickly washed with Pannett-Compton solution and placed in a watch glass over a small pool of ice-cold fixative; fixative was also used to flood the inside of the glass ring and submerge the embryo. The assembly was kept on ice for 30 min. Dissected embryos were brought into absolute ethanol, embedded in paraffin and serially cut at 5 µm. Sections were placed on metal-framed, 0.9 µm thick POL membranes (cat. no. 11505191; Leica), and the regions of interest were dissected using a Leica LMD 600 laser capture microdissection system fitted with a ×63 lens and fluorescence optics. The total RNA was quality-checked on a pico chip (Bioanalyzer 2100; Agilent), amplified with SMARTer kit (Clontech), and sequenced on an Illumina HiSeq 4000 platform (100 bp runs, pair end).

Gene counts and comparisons of gene expression levels between each pair of samples were performed using the Trapnell pipeline [35]. For each pair, we assigned a score of 1 to genes at least five times more expressed in the ‘red’ sample (from the MSZ) than in the ‘white’ one (from surrounding regions), and a score of 2 if there was a significant gene count in the ‘red’ sample but zero in the ‘white’ one. Genes were then ranked based on the aggregate score (electronic supplementary material, table S1, sheet 1). For further assessment by RT-PCR (electronic supplementary material, table S1, sheet 2), we discarded the genes whose expression patterns were known not to be specific to the regions of interest, but included low-ranking genes showing very dissimilar levels in at least some pairs of samples.

## Data Availability

The RNA-seq data have been deposited in the Gene Expression Omnibus under GSE107928 [36]. Supplementary material is available online [37].
